# Localised Infection of Atlantic Salmon Epithelial Cells by HPR0 Infectious Salmon Anaemia Virus

**DOI:** 10.1371/journal.pone.0151723

**Published:** 2016-03-21

**Authors:** Maria Aamelfot, Debes H. Christiansen, Ole Bendik Dale, Alastair McBeath, Sylvie L. Benestad, Knut Falk

**Affiliations:** 1 Norwegian Veterinary Institute, Oslo, Norway; 2 Food and Veterinary Authority, Tórshavn, Faroe Islands; 3 Marine Scotland Science, Marine Laboratory, Aberdeen, Scotland; Friedrich-Loeffler.Institut, GERMANY

## Abstract

Infectious salmon anaemia (ISA) is an important, systemic viral disease of farmed Atlantic salmon, *Salmo salar* L. Endothelial cells are the main target cells for highly virulent HPR-deleted ISA virus (ISAV) types. Here we examine the pathogenesis of non-virulent ISAV HPR0 infections, presenting evidence of an epithelial tropism for this virus type, including actual infection and replication in the epithelial cells. Whereas all HPR0 RT-qPCR positive gills prepared for cryosection tested positive by immunohistochemistry (IHC) and immunofluorescent labelling, only 21% of HPR0 RT-qPCR positive formalin-fixed paraffin-embedded gills were IHC positive, suggesting different methodological sensitivities. Only specific epithelial cell staining was observed and no staining was observed in endothelial cells of positive gills. Furthermore, using an ISAV segment 7 RT-PCR assay, we demonstrated splicing of HPR0, suggesting initial activation of the replication machinery in the epithelial gill cells. Immunological responses were investigated by the expression of interferon-related genes (*e*.*g*. Mx and γIP) and by ELISA for presence of anti-ISAV antibodies on samples taken sequentially over several months during an episode of transient HPR0 infection. All fish revealed a variable, but increased expression of the immunological markers in comparison to normal healthy fish. Taken together, we conclude that HPR0 causes a localized epithelial infection of Atlantic salmon.

## Introduction

Infectious salmon anaemia virus (ISAV) is the causative agent of a systemic and lethal disease (ISA) in farmed Atlantic salmon, *Salmo salar* L. The virus belongs to the genus *Isavirus* of the family *Orthomyxoviridae*. Clinical signs suggest circulatory failure with severe anaemia, ascites, congestion and enlargement of the liver and spleen (reviewed in [1]). Endothelial cells are the main target cells for virulent virus replication [2], however epithelial cells may also be infected in early stages, especially by low virulent isolates [3, 4]. Immersion infection experiments suggest variations in replication dynamics in gill [5] and other mucosal surfaces [4] between ISAV strains of low and high virulence, with subsequent variations in disease severity and pathogenesis [6].

Putatively non-virulent genotypes of the virus (ISAV HPR0) have been detected in all major Atlantic salmon producing countries including Norway [7], Scotland [8, 9], Canada [10], Chile [11] and the Faroe Islands [12], but is not associated with clinical ISA disease. HPR0 is differentiated from the low and highly virulent types based on a ‘full length’ highly polymorphic region (HPR) located upstream of the trans-membrane region of genomic segment 6 encoding the HE protein [8, 13, 14]. Even though additional changes are likely required for the transformation, the main theory is largely accepted that virulent ISAV strains have arisen following various deletions within the HE gene of HPR0 types. This suggests the HPR0s are ancestors of virulent HPR-deleted (HPR-del) ISAV strains.

After a devastating ISA disease epidemic in the Faroe Islands, the Atlantic salmon farming industry was re-established and the risk for re-emergence of ISA and ISAV was investigated [12]. During the study period, 11.9% of gills from apparently healthy Atlantic salmon tested positive for HPR0 by real-time RT-PCR. One particular year, HPR0 was associated with all Atlantic salmon production cycles, appearing highly infectious [12]. The infection was seasonal and transient, and the virus prevalence and level was substantially higher in gill compared to kidney [12] indicating a different tropism to that of the low and highly virulent HPR-del ISAV types. Random anonymous surveillance for HPR0 in Scotland detected viral material in gills from healthy salmon in 11% of sites tested [9], and a comparable prevalence is reported from both Norway and Chile [7].

HPR0 has yet to be cultured in the laboratory, making infection experiments problematic. Given the short-lived and transient nature of the infection, in addition to the lack of clinical signs in the field, good samples from infected fish are difficult to obtain. Thus, the samples collected in the Faroe Islands, represent an important and unique resource for HPR0 investigation. To better understand the specific tropism and pathogenesis of HPR0 we studied the infection using selected tools from work with virulent ISAV, including immunohistochemistry, immunofluorescence, ELISA and RT-qPCR.

## Materials and Methods

### Organ sampling

The organ samples were collected by veterinary inspectors at the Faroese Food and Veterinary Authority (FFVA), as part of an official, mandatory surveillance program for fish diseases in Faroese Atlantic salmon aquaculture. All samplings were performed in accordance with Faroese regulations on animal welfare, and included anaesthetization using Finquel^®^Vet, followed by a blow to the head to kill the fish before sampling.

The selected farms experienced episodes of high HPR0 loads around the time of sampling. Gills were collected from 80 fish at farm I (in April 2007) and 40 fish at farm II (in December 2007) in 10% phosphate buffered formalin and in 500 μl RLT-lysis buffer (Qiagen). In addition, kidney samples were also collected from farm II. At farm III, gills from 280 fish were collected in RLT-buffer over a 4-month period (from August to November 2009), (41, 80, 79 and 80 fish each month respectively) coinciding with an HPR0 infection cycle. In addition, plasma was collected at farm III for ELISA from 64 fish across the same 4 months (18, 6, 20 and 20 fish each month respectively). Gills were collected at farm IV (in February 2010) from 20 fish in RLT-buffer. In addition, gills were frozen on dry ice (without fixation) and gill swabs were collected in 500 μl RLT buffer. From farm V (in February 2014), gills and fin swabs were collected in RLT buffer from 19 fish. The formalin fixed samples were embedded in paraffin and processed by standard histological procedures and sections were stained with haematoxylin and eosin. Routine histological examination was performed. Frozen samples were sectioned on a cryotome and stored at −80°C until immunostaining.

### Immunohistochemistry

Immunohistochemistry (IHC) for ISAV was performed as previously described [15] on formalin fixed paraffin embedded gills from farm I and II, and on frozen gills from farm IV. Briefly, polyclonal rabbit antibody to recombinant ISAV nucleoprotein (NP) [16] was used for the virus detection. Vectastain ABC-AC kit (Vectastain anti rabbit Ig ABC-AP kit, AK 5001, Vector Laboratories, Inc.) was used for detection of bound polyclonal antibody using Fast Red (1 mg ml^-1^) and Naphtol AS-MX phosphate (0.2 mg ml^-1^) with 1 mM Levamisole in 0.1 M TBS (pH 8.2) as a substrate. In addition, a monoclonal antibody to ISAV haemagglutinin esterase (HE) [17] was used for the virus detection on the cryosections. An HRP conjugated anti-mouse Ig amplified detection system (EnVision+, Dako) was used for detection of bound monoclonal antibodies using 3,3’-diaminobenzidine (DAB) as substrate. All sections were counterstained with haematoxylin.

### Immunofluorescent antibody test

Immunofluorescent antibody test (IFAT) for ISAV was performed as previously described [17] on frozen gills from farm IV. Briefly, sections of frozen gills were fixed in 80% cold acetone for 10 min. A polyclonal rabbit antibody to recombinant ISAV NP [16], or a monoclonal antibody to ISAV HE [17] was used for virus protein detection. Alexa Fluor^®^ 594 conjugated anti rabbit IgG (Molecular Probes) and Alexa Fluor^®^ 488 conjugated anti mouse IgG was used for detection of bound antibody, and sections were mounted in SlowFade^®^ Gold (Molecular Probes).

Double immunofluorescent staining was performed with antibody to ISAV HE and monoclonal antibody to either Atlantic salmon endothelial cells [18] or polyclonal antibody to cytokeratin (AE1/AE3 (Sigma)) detecting endothelial and epithelial cells respectively as previously described [3, 18]. Alexa Fluor^®^ secondary antibodies (Molecular Probes) were used for detection of bound antibodies, as described above.

### Real-time RT-PCR and PCR

#### ISAV segment 8 real-time RT-PCR

Total RNA was extracted from organ samples or from gill swabs from all farms using the RNeasy 96 mini kit (Qiagen) following manufacturers protocols with minor modifications. Subsequently a duplex ISAV real-time RT-PCR (RT-qPCR) assay was used for detection of ISAV segment 8 and the endogenous control, elongation factor-1α (ELF) using the QuantiTect Probe RT-PCR kit (Qiagen) as previously described [12]. Primers and probe were used for ISAV [12] and ELF [19] as reported previously.

To investigate potential loss of virus and virus infected cells during preparation for histology 14 gills from farm II were re-tested post-formalin fixation and paraffin embedding. Total RNA was extracted from the paraffin embedded gill tissue blocks using the RNeasy FFPE kit (Qiagen) as recommended by the producer and the presence of ISAV was tested with the ISAV duplex RT-qPCR assay described above.

#### Immune markers—real-time RT-PCR

RNA from 15 selected RT-qPCR HPR0 positive samples from farm II including IHC positives and negatives was extracted, reverse transcribed and analysed by RT-qPCR using primers and probes for the immune markers Type I and Type II interferon (IFN), Mx and γIFN-induced protein (γIP) and the endogenous control ELF as described previously [5]. Results are presented as ratios compared to negative control fish data from a recent infection trial used to provide a baseline for unexposed fish [6]. This was not ideal due to the substantially different sample backgrounds between aquarium trial and farm samples nevertheless we felt a baseline comparison was prudent.

RNA species specific reverse transcription (RT) and qPCR was performed on the 15 selected samples as described previously [20] utilising tagged primers adapted for low and highly virulent strains [5]. The method was only used to indicate presence or absence of the specific RNAs. In addition, two-hundred samples (months 1–3) from farm III were analysed by two step RT-qPCR for expression of immune markers Mx and γIP in response to an HPR0 infection as previously described [21].

#### ISAV segment 7 PCR

An RT-PCR assay using the one-step RT-PCR kit (Qiagen) was developed to individually distinguish the two ISAV segment 7 open reading frames (ORF) generated by alternative splicing [22]. The reaction mixture was as recommended by the manufacturer including the ISAV segment 7 specific forward: 5’-agctaagattctccttctacaatgg-3’ and reverse: 5’-ccttcaagaggtccagcatacc-3 primers (DNAtechnology) which generated a 692 base pair (bp) product for ORF 1 and a 166 bp product for ORF 2. The RT-PCR was performed with an ABI 2710 Thermocycler (Applied Biosystems) using the following conditions: cDNA synthesis at 50°C for 30 min, activation of HotStart Taq polymerase at 95°C for 15min and 40 cycles of 94°C for 30 s, 60°C for 30 s and 72°C for 60 s. Following amplification the PCR products were separated on the Agilent 2100 bioanalyzer (Agilent) using the DNA 1000 kit as recommended by the manufacturer.

Infected cell culture supernatant was collected from ISAV-infected Atlantic salmon kidney cells. After complete cytopathogen effect (CPE), the supernatant was filtered through a 0.22 μM Wattman filter before RNA was extracted and subjected to the segment 7 specific RT-PCR as described above.

### ELISA

ELISA was used to determine ISAV-specific antibodies in salmon plasma from farm III as previously described [23]. Briefly, antigens for the ELISA were prepared from cell lysates of ISAV-infected and mock-infected SHK-1 cells. Polystyrene microtitre plates (Immuno-Plate Maxisorp, Nunc) were coated overnight at 4°C with 100 μl per well of either ISAV-, or control antigen preparation diluted to 2.5 μgml^-1^ in 50 mM sodium carbonate buffer, pH 9.6. After washing and blocking with 5% dry milk in 0.1% PBS Tween 20 for one hour, plasma diluted 1:200 were added in duplicates and incubated at 4°C overnight. Polyclonal rabbit anti-salmon Ig and subsequently peroxidase conjugated anti-rabbit Ig (Amersham Pharmacia, Uppsala, Sweden) were added in dilutions of 1:1000 in 0.1% PBS Tween 20 and incubated at room temperature for 60 min and 45 min, respectively. Plates were incubated with a mixture of substrate buffer (0.015% H_2_O_2_, 4% DMSO and chromogen TMB (Bio-Rad)). The reaction was stopped with 1N H₂SO₄ (Bio-Rad) after 20 min. Readings were measured as optical density (dual filter 450/620 nm). Values of the control antigen were subtracted from results of the ISAV-antigen to eliminate background of the coating antigen itself. A pool of five plasma samples from natural ISAV-infected, convalescent fish and of five healthy, non-immunized fish were used as positive and negative controls, respectively. The test results were presented as a percentage of the positive control run on each plate. Test-plasma with a value ≥10% compared to the positive control reading were regarded as positive.

## Results

### Autopsy findings and histology

No clinical signs of ISA, anaemia, nor incidents of increased mortality were detected in any of the fish from any of the farms. Gills from ISAV HPR0 infected farms were selected randomly for histological examination. All available kidney samples were investigated. No major cellular inflammatory cell responses nor parasites or bacteria were observed by histological evaluation. Focal, moderate hypertrophy and hyperplasia of the lamellar epithelium were seen in some gills. These findings support that HPR0 infections do not lead to any ISA-like disease.

### IHC and IFAT

**Fig 1:**
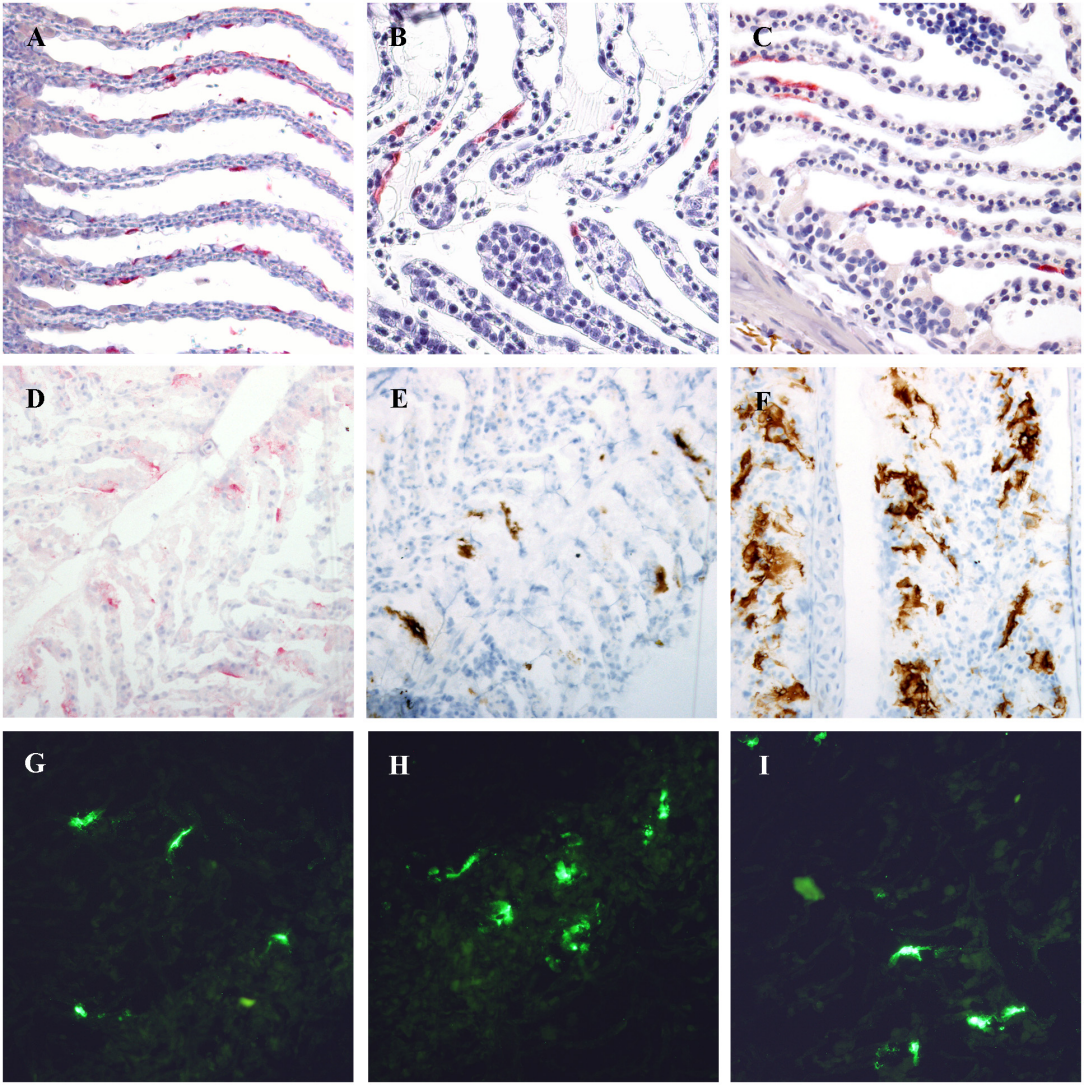
Immunostaining of tissue sections. Immunostaining of formalin-fixed paraffin-embedded (A-C), and frozen (D-I) gill sections with massive and sparse infection ISAV HPR0 infection, demonstrating epithelial infection. Primary antibodies used were a rabbit antibody to ISAV NP (A-D, G), and a monoclonal antibody to ISAV HE (E-F, H-I). For detection of bound antibodies, alkaline phosphatase-conjugated antibody to rabbit Ig with Fast Red substrate (A-D; red colour), horseradish peroxidase-conjugated antibody to mouse Ig with DAB substrate (E-F; brown colour), and Alexa fluor 488-conjugated antibody to rabbit Ig (G) or mouse Ig (H-I), were used.

Formalin fixed paraffin embedded gills from farm I revealed one IHC positive fish out of the 80 fish tested. From farm II, 17 of 40 fish were positive (S1 Table). In the positive fish, IHC revealed strong positive ISAV staining confined to the epithelial cells (Fig 1A–1F). Viral dissemination from epithelial cells to adjacent endothelial cells was not found, even in gills with low Ct-values (Ct < 20) by RT-qPCR. The location of the ISAV nucleoprotein in the epithelial cells was most frequently present in the cytoplasm, but in some cells confined to the nucleus, in keeping with the orthomyxoviral replication cycle. All kidneys tested (Ct value range 31.4–36.5 [12]) were negative by IHC.

All frozen gills from farm IV were positive by IFAT using antibody to ISAV HE (Fig 1H and 1I). scoring from 1 to 3 (of maximum 3) using our previously established IHC scoring system [6] while 16 of 20 were positive using antibody to ISAV NP (Fig 1D and 1G, Table 1). Freezing of samples on dry ice does not give optimal morphology, however by combining double IFAT staining for endothelial and epithelial cells we were able to counterpart the poor morphology and were able to distinguish the cell types in the sections. Double staining with antibody to cytokeratin confirmed that positive cells were epithelial cells. Double staining with antibody to endothelial cells showed no overlap, *i*.*e*. no endothelial cells were infected in the frozen sections (data not shown).

**Table 1:** RT-qPCR of gills and gill swabs, and immunostaining on frozen gills from farm IV.

Fish	Gill sample ISAV	Gill sample ELF	Gill swab ISAV	Gill swab ELF	HE IFAT	HE IHC	NP IFAT	NP IHC
1	27.76	20.86	21.21	20.71	3	3	3	3
2	20.08	19.31	22	21.28	3	3	3	1
3	23.52	19.58	24.13	19.27	3	2	1	1–2
4	20.82	19.45	20.66	19.79	3	3	2	2–3
5	20.85	20.19	21	19.88	3	2	2	1
6	22.83	19.06	25.05	21.46	2–3	3	2	1
7	20.13	18.56	21.9	20.09	3	3	3	1
8	23.4	18.17	26.02	20.06	1	1	1	1–2
9	19.49	18.76	20.75	19.68	3	3	3	1
10	25.1	19.56	25.75	19.94	2	1	1	1
11	34	20.45	34.34	20.11	1	3	-	-
12	32.29	20.47	30.53	20.54	1	-	-	-
13	25.45	19.56	25.68	20.15	2	2	2	1
14	30.06	19.68	26.69	20.59	1	-	1	1
15	23.59	18.31	25.24	20.56	3	1	1	-
16	28.26	19.66	28.62	20.77	1	-	-	-
17	28.45	19.49	29.01	20.91	1	1	-	-
18	28.64	20.41	29.17	20.2	1	1	1	1
19	27.34	20.61	25.58	18.78	1	1	1	-
20	28.6	20.03	29.27	20.14	1	1	1	-
	**25.53**	**19.61**	**25.63**	**20.25**	**20**	**17**	**16**	**14**
	**Mean**				**Number of positive fish**			

ISAV segment 8 and ELF RT-qPCR Ct-values from gill sample and gill swabs from farm IV. IHC and IFAT score from 1 to 3 (of maximum 3) on frozen gills. -: negative.

IHC on frozen gills using antibody to ISAV NP produced epithelial labelling in 14 of 20 gills (Table 1). In some samples, labelling was detected extracellularly and we speculate whether the freezing process combined with acetone fixation may have dissolved the antigen (Fig 1E and 1F). IHC on frozen gills using antibody to ISAV HE produced a more distinct epithelial cell-bound or cell-associated labelling (Fig 1E and 1F) with labelling in 17 of 20 fish (Table 1). A distinct pattern revealed large amounts of positive cells especially in multi-layered epithelium. In addition, several positive epithelial cells were detected in the lamellae, both basal cells and cells more proximal in the lamellae of the heavily infected fish. In a few gills, almost all the lamellar epithelial cells were labelled.

In summary, immunostaining on frozen sections from farm IV gave a higher prevalence (20 of 20) of positive fish compared to IHC on formalin fixed paraffin embedded sections (17 of 40) from farm II.

### ISAV RT-qPCR in gills and swabs

The number of positives, mean Ct-values and range of ISAV segment 8 RT-qPCRs from gills from farms I to V are listed in Table 2. A high frequency of RT-qPCR positive gills was found in all five farms. The Ct-values were often very low indicating large amounts of virus. RT-qPCR gave a higher prevalence of positives compared to IHC. The mean Ct-value for the IHC positive fish from farm II were approximately 5 cycles lower than the mean Ct-value for the IHC negative fish from the same farm (Table 2).

**Table 2:** Summary of RT-qPCR on gills.

Farm	Mean Ct	Range	No. positives
**I**	31.16	18.7–36.1	58 of 80
**II—IHC pos**	22.91	18.6–27.6	40 of 40
**II—IHC neg**	27.69	20.9–33.1	40 of 40
**III a**	na	na	0 of 41
**III b**	31.6	20.8–36.9	58 of 80
**III c**	35.6	34.2–36.5	4 of 79
**III d**	36.3	na	1 of 80
**IV**	25.53	19.5–34.0	30 of 30
**V**	32.3	20.6–36.1	11 of 19

ISAV segment 8 RT-qPCR mean Ct-values, range and prevalence of positive gill samples from farms I to V. IHC pos: corresponding formalin fixed paraffin embedded samples positive for ISAV NP IHC. IHC neg: corresponding samples negative for ISAV NP IHC, na: not applicable, a: August, b: September, c: October, d: November.

RT-qPCR testing of RNA extracted from formalin-fixed paraffin-embedded sections revealed a 100–10,000 fold increase in Ct-values for ISAV segment 8 and ELF compared to testing RNA extracted from samples in RLT-buffer. *I*.*e*. an increase of 5–10 cycles (mean 8) for ELF and 6–13 cycles (mean 10) for ISAV segment 8 (data not shown) indicate a substantial reduction in target nucleic acid. This may indicate a loss of infected cells during the fixation and embedding process, perhaps explaining the lower level of HPR0 positives detected by IHC than by IFAT and RT-qPCR. All samples were ISAV positive pre-fixation. Only 10 of 14 samples were ISAV positive post-fixation.

The ISAV segment 8 (and ELF) RT-qPCR Ct-values from gill samples (mean Ct 24.30, range 18.61–34.00) and gill swabs (mean Ct 25.63, range 20.66–34.34) from farm IV are listed in Table 1. In many fish, we found comparable Ct-values in the gill samples and gill swabs.

The Ct-values from gills samples (mean Ct 32.28, range 20.55–36.1) and fin swabs (mean Ct 32.86, range 27.23–35.96) from farm V are listed in Table 3. All fish positive in the gill samples from farm V were positive in the fin swabs (11 of 19), however three fish with positive fin swabs were negative in the gills. The ELF Ct-values indicate presence of viable cells in the swabs. These results suggest that HPR0 also infect fin/skin epithelial cells.

**Table 3:** RT-qPCR on gills and fin swabs.

Fish	Gill sample ISAV	Gill sample ELF	Fin swab ISAV	Fin swab ELF
1	-	19.04	34.85	20.12
2	34.29	18.65	32.1	20.44
3	-	20.16	32.71	20.37
4	-	19.26	35.14	22.2
5	36.1	19.56	34.05	22.12
6	-	19.17	-	23.01
7	33.77	19.03	34.37	19.11
8	35.73	17.42	35.96	19.39
9	-	18.12	-	20.92
10	-	18.46	-	21.95
11	36	18.38	33.16	19.65
12	-	18.56	33.05	18.28
13	30.44	19.17	27.23	19.6
14	20.55	17.75	33.41	19.15
15	28.55	19.11	30.75	20.75
16	36.01	21.04	33.58	19.98
17	29.89	18.89	29.84	16.6
18	33.78	18.05	33.04	17.36
19	-	18.37	32.59	17.34
**Mean:**	**32.28**	**18.85**	**32.86**	**19.91**
**No. pos:**	**11**		**16**	

ISAV segment 8 and ELF RT-qPCR Ct-values from gills and fin swabs from farm V.–: negative. No. pos: Number of positive fish.

**Fig 2:**
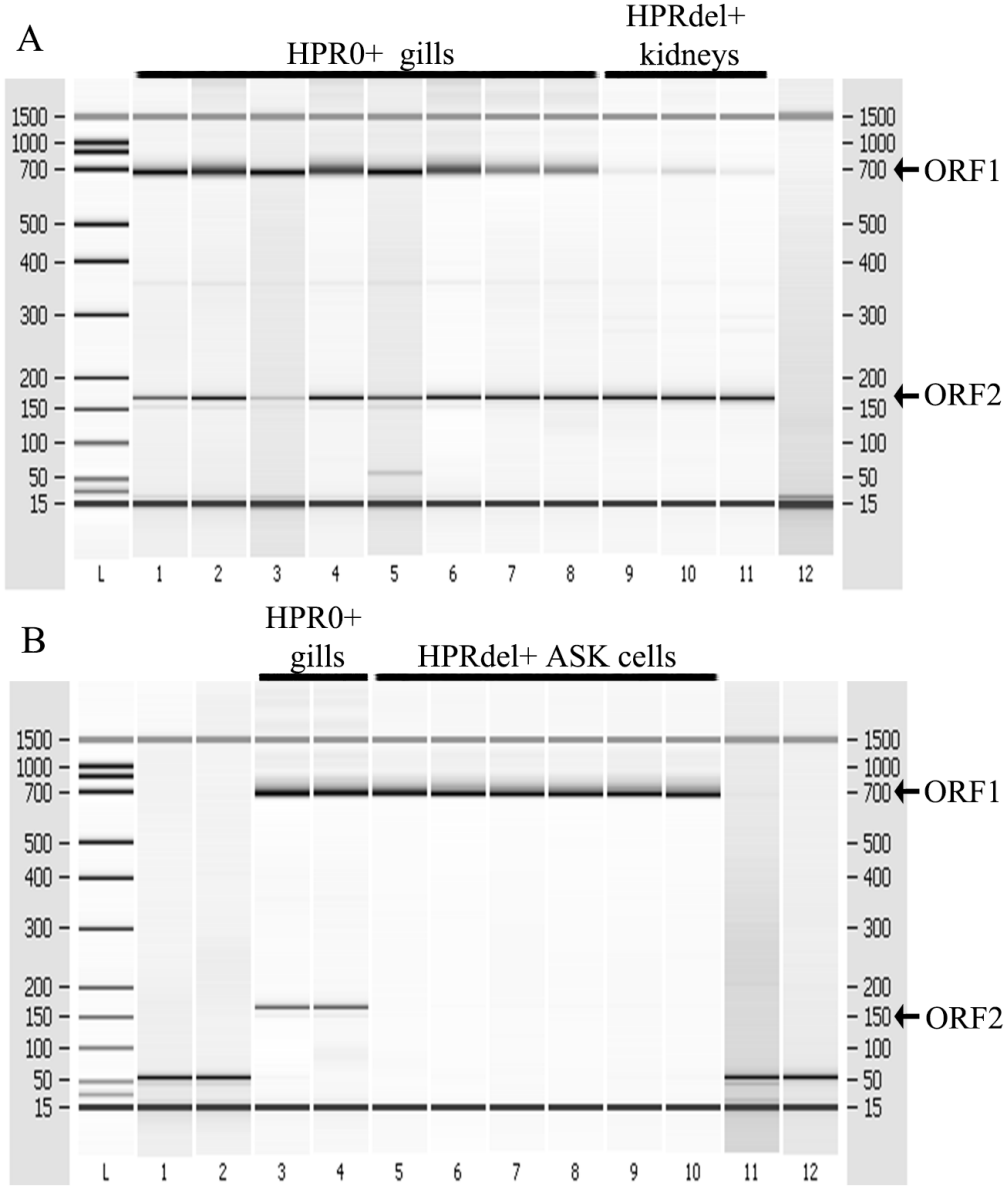
Demonstration of splicing by RT-PCR analysis of HPR0 infected gill, HPR-del infected kidney, and HPR-del infected ASK cell cultures. Segment 7 RT-PCR demonstrating successful amplification of the two ORFs in HPR0 positive gills and HPR-del positive kidneys A. Segment 7 ORF2 is produced following a splicing event and is therefore a definitive indicator of active transcription of HPR0 in gill and kidney cells. B. Lack of ORF2 demonstrates that only viral particles are present in the supernatant of infected ASK cell cultures.

The segment 7 RT-PCR designed to demonstrate splicing, revealed successful amplification of the two overlapping ORFs, suggesting an active transcription in HPR0 infected gills (Fig 2A and 2B) from farm II and in fin swabs from farm V (data not shown). The in-frame splicing was documented by direct sequencing of the ORF1 and ORF2 RT-PCR products (data not shown).

This was further substantiated by the RNA subspecies specific assay detecting both mRNA and cRNA in 9 of 15 samples from farm II while mRNA only was detected in a further 2 samples, demonstrating both transcription and active replication. All samples with high ISAV segment 8 Ct-values (Ct > 30) or samples negative by RT-qPCR were negative by the RNA specific assays.

### Immune markers in gills

Fifteen HPR0 infected gill samples from farm II randomly selected for immune marker expression showed increases in all markers tested when compared to uninfected baseline data obtained from negative fish of an experimental challenge in 2012 [5, 6] (Table 4) providing indication of a host immune response during HPR0 infection. High increases of type II related products were particularly notable, γIFN was 14–46 fold higher and γIP was 10–100 fold higher. As expected [5], considerable between fish variation, not correlated with the level of detected virus, was seen.

**Table 4:** Immune marker RT-qPCR.

Fish	ISAV seg8	Type 1 Mx	Type 1 a/bIFN	Type 2 γIP	Type 2 gIFN
2	20.03	1.86	3.70	15.98	15.16
3	21.27	1.16	3.15	32.29	30.86
7	28.36	8.34	7.50	104.37	46.29
13	20.79	3.85	3.12	37.28	29.27
16	20.40	2.26	3.27	27.22	33.50
17	31.96	1.49	2.86	14.15	35.55
18	19.02	2.11	3.93	79.74	43.80
23	21.61	1.54	1.73	20.39	20.67
26	20.65	1.65	1.36	41.59	37.58
28	27.11	3.05	3.79	29.54	39.28
29	20.63	1.95	4.22	39.83	32.83
32	23.84	2.35	3.05	31.36	21.78
33	25.48	1.15	1.57	9.94	16.82
35	23.52	8.37	6.73	39.31	30.15
40	21.14	1.28	2.04	16.64	14.02

ISAV segment 8 RT-qPCR Ct values and immune marker expression ratios of gills from 15 fish from farm II compared to an uninfected baseline.

**Fig 3:**
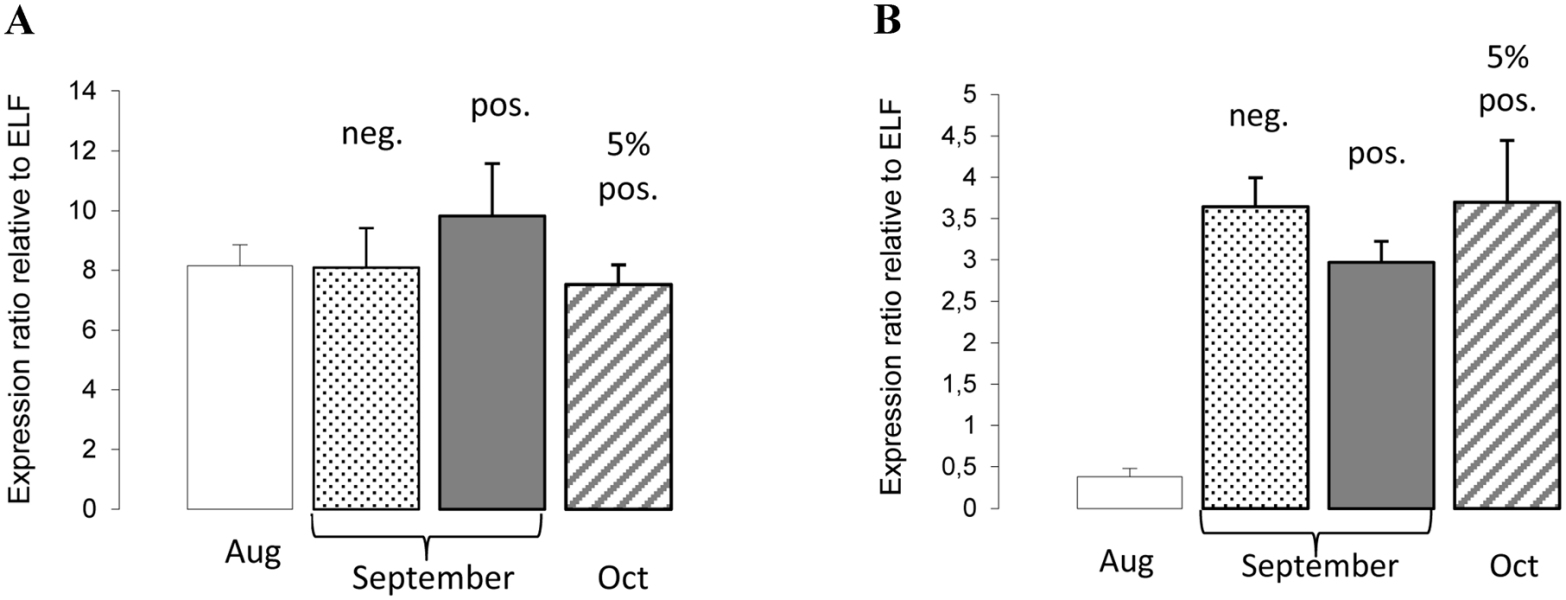
Mx and γIP response to HPR0 infection at farm III. Across the three months, little variation in Mx expression (A) was seen. However, γIP was over 8-fold lower in the fish sampled in August compared to those sampled in September and October (B), suggesting the presence of a γIFN-induced response. In August 2009, all fish (n = 41) tested negative (neg.) for HPR0. In September 2009, 72% (58 out of 80) fish tested positive (pos.) (mean Ct 31.5, range 20.8–36.7). In October 2009, 5% (4 out of 79) fish tested positive (mean Ct 35.6, range 34.2–36.5). Ratios standardised relative to elongation factor 1α (ELF).

The response of two immune markers, Mx and γIP was investigated in gills from farm III sampled during the first 3 months of the HPR0 infection. In August 2009, all fish (n = 41) tested negative for HPR0 by RT-qPCR. The following month (September 2009) 58 out of 80 fish tested positive (mean Ct 31.5, range 20.8–36.7). In October 2009, 4 out of 79 fish tested positive (mean Ct 35.6). Across all three months, little variation in Mx expression was observed (Fig 3A). However, γIP was over 8-fold lower in the fish sampled in August compared to those sampled in September and October (Fig 3B), suggesting the presence of a γIFN-induced response.

### ELISA

The antibody responses in 64 fish from farm III and the equivalent ISAV segment 8 RT-qPCR results are summarised below. In August 2009, all fish tested negative for HPR0 by RT-qPCR, however 3 of 18 (16.67%) were positive for anti-ISAV antibodies. The following month (September) 2 of 6 (33.33%) were positive for antibodies while all (100%) tested positive for HPR0 by RT-qPCR (mean Ct 30.1). In October, all 20 fish tested negative for HPR0, while 9 of 20 (45%) tested positive for antibodies. In November, all 20 fish tested negative for HPR0 while 2 of 20 (10%) tested positive for antibodies. The results suggest an induction of a short-lived specific antibody response.

## Discussion

Here we demonstrate that ISAV HPR0 causes a localized and superficial epithelial infection in mucosal surfaces of the gills and skin of Atlantic salmon. This virus type does not infect endothelial cells commonly infected by virulent ISAV HPR-del strains. The results also suggest, though only a few immunological parameters were examined, that immune responses associated with these infections may be limited. We present valuable data from healthy Atlantic salmon from five independent HPR0 positive farms located in the Faroe Islands providing unique information on HPR0 infections in the general environment.

Using IHC and IFAT, we found that HPR0 appears epitheliotropic, and does not cause a systemic endothelial infection, even in fish with large amounts of virus (RT-qPCR Ct-values approx. 20). Interestingly, we recently showed that the skin and fins, in addition to the gills, are important for uptake and early infection of virulent strains of ISAV in experimentally infected Atlantic salmon [4]. In the present study, detection of HPR0 in epithelial swabs from the fin/skin with higher prevalence than in the gills, indicate HPR0 is not confined to the gills either, suggesting skin is also an important target for HPR0 infections. Swabbing is a non- lethal sampling method and could thus be used for screening for the presence of HPR0 in farmed fish.

Epithelial infection has never been reported in Atlantic salmon from ISA outbreaks in the field, where endothelial infection is a constant finding [2]. Interestingly, positive epithelial cells have been detected in fish experimentally infected with virulent strains of ISAV [2, 3, 4]. A low virulent strain of ISAV had higher affinity to epithelial cells than a highly virulent strain, even though both also replicated in endothelial cells and caused viremia and a systemic infection [5, 6], suggesting the low virulent strain has similar properties as the HPR0.

The initial low prevalence of HPR0 positive gills by IHC compared to by RT-qPCR, led to queries regarding the sensitivity and specificity of the methods used. In addition, we speculated whether RT-qPCR positive gills and epithelial swabs could in fact be virus picked up from the environment, *i*.*e*. not truly replicating virus. Mucosal surfaces are covered by mucus, a source of sialic acids potentially containing the specific ISAV sialic acid receptor [2, 24]. Therefore, the mucosal surface could serve as a virus enrichment system. To examine this possibility, we firstly investigated if virus replication was actually occurring at the mucosal surfaces. Transcription was indicated by RT-qPCR detection of segment 8 mRNA and confirmed by the RT-PCR amplification of the segment 7 spliced ORF2 product, providing strong evidence of active transcription of HPR0. Viral replication was confirmed by RT-qPCR detecting cRNA, the replicative intermediary only present during active viral replication cycles [20], and by protein production indicated by immunostaining detecting NP- and HE-proteins. Taken together, these results confirmed active infection and replication by HPR0.

Secondly, in addition to a general lower sensitivity of IHC compared to RT-qPCR [6], we investigated whether the discrepancy in prevalence could be caused by loss of infected epithelial cells during the histological preparation process. Formalin fixed paraffin embedded samples were retested by RT-qPCR, which produced higher Ct-values compared to samples directly lysed in RLT-buffer indicating that this removal occurred. IHC and IFAT on frozen gill samples (not subjected to formalin fixation and paraffin embedding) produced a much higher prevalence of positive samples compared to IHC on formalin fixed paraffin embedded samples confirming our suspicion.

A major difficulty with samples obtained from the general environment, in addition to the timing and dose of infection, is the uncontrolled nature of exposure to other factors that may influence the results. Even in experimental challenges where these factors are controlled, variation in viral loads and immune responses between individuals may still be considerable [5]. In this study, a transient immune response to the HPR0 virus was found. At farm III, an increased adaptive/type II IFN response, indicated by higher γIP expression and antibody detection, correlated with detection of the virus in September and October. A major function of γIP is T-cell stimulation [25]. Previously, viruses, including viral haemorrhagic septicaemia virus and ISAV, have been shown to regulate γIP expression in fish [5, 21, 26, 27]. Furthermore, no significant difference in the antiviral protein Mx was found. All fish were negative for ISAV in August, however they may have been in the early stages of infection whilst the virus was at undetectable levels thus prompting an early response. Similarly, whilst only 5% of the fish tested were positive for HPR0 in October, most had likely been previously exposed to the virus thus producing the increased γIP induction, indicating a γIFN-induced type II response even in the negative fish. Finally, the high induction of both γIFN and γIP in the 15 fish from farm II tested for immune marker expression provided another indication of a strong type II response, but this needs to be further investigated.

HPR0 infection episodes are common in Atlantic salmon farming and published results suggest that most salmon populations experience this infection during their life cycle [7, 12]. Whether an HPR0 infection could provide any immunological protection against repeated infections, either with HPR0 or HPR-del virus, has been questioned. Our results suggest a limited response following HPR0 infections, however if this response is sufficient to protect against repeated HPR0 infections or against classical HPR-deleted virulent strains is still not known. In Norway, HPR0 was detected on a farm only months prior to a confirmed ISA diagnosis [28]. Furthermore, repeated HPR0 detections have been seen in farms in the Faroe Islands [12]. Taken together HPR0 infections do not appear to induce a substantial level of protection against either virus type.

Previously, we indicated similarities between ISAV infection in Atlantic salmon and highly pathogenic influenza (HPAI) infections, including the endotheliotropic nature of both viruses [2]. Here we provide information indicating similarities between HPR0 and low pathogenic influenza (LPAI) which also has epithelial cells as their main target. Cell and organ tropism (*i*.*e*. epithelium vs. endothelium) depend on receptor availability and on fusion activity. The ability of virulent ISAV and HPAI to spread systemically has both been associated with presence of the appropriate fusion activation protease [29] and polymerase activities however the significance of the polymerases for ISA pathogenesis remains to be elucidated. HPR0 and LPAI do not appear to spread systemically, but are largely contained at the epithelial surface. In addition, HPR0 does not replicate in cell cultures, similar to LPAI, which does not replicate in cell cultures without addition of an external protease. To substantiate this comparison further knowledge about ISAV in relation to influenza virus infections is required.

We conclude that HPR0 causes a localized epithelial infection of Atlantic salmon gills capable of stimulating an immune response the extent of which remains to be further elucidated. Improving our understanding of the virus tropism and pathogenesis of HPR0 is crucial to understand the risk of this non-virulent virus changing into a highly virulent type and the impact HPR0 has on the Atlantic salmon farming industry.

## Supporting Information

S1 Table RT-qPCR of gills and IHC on formalin fixed paraffin embedded gills from farm II. ISAV segment 8 and ELF RT-qPCR Ct values and IHC using antibody to ISAV NP on gills from farm II. All fish were positive for HPR0 by RT-qPCR, but only 17 of 40 were positive by IHC. +: positive labelling of epithelial cells in the gill. -: negative, nt: not tested.
(PDF)

## Abbreviations

Bp: base pair
ELF: elongation factor-1α
HE: haemagglutinin esterase
HPR: highly polymorphic region
HPR0: putatively non-virulent variants of ISAV
IFAT: immunofluorescent antibody test
IHC: immunohistochemistry
IFN: interferon
ISA: infectious salmon anaemia
ISAV: infectious salmon anaemia virus
NP: nucleoprotein
ORF: open reading frame
RT: reverse transcription
γIP: γIFN-induced protein
