# Supplementary material for: Localised Infection of Atlantic Salmon Epithelial Cells by HPR0 Infectious Salmon Anaemia Virus
Source: PLoS One. 2016 Mar 21;11(3):e0151723. doi: 10.1371/journal.pone.0151723 (PMC4801213; doi:10.1371/journal.pone.0151723)
Supplement: S1 Table — ISAV segment 8 and ELF RT-qPCR Ct values and IHC using antibody to ISAV NP on gills from farm II. All fish were positive for HPR0 by RT-qPCR, but only 17 of 40 were positive by IHC. +: positive labelling of epithelial cells in the gill. -: negative, nt: not tested. (PDF) [file pone.0151723.s001.pdf]

Table S1. RT-qPCR of gills and IHC on formalin fixed paraffin embedded gills from farm II. ISAV segment 8 and ELF RT-qPCR Ct values and IHC using antibody to ISAV NP on gills from farm II. +, positive labelling of epithelial cells in the gill. -, negative. Nt: not tested. All fish were positive for HPR0 by RT-qPCR, but only 17 of 40 were positive by IHC.

Fish	ISAV Ct	ELF Ct	Gill IHC
1	30.02	20.47	-
2	20.03	19.30	+
3	21.75	19.63	+
4	18.57	nt	+
5	26.97	19.68	-
6	33.06	19.62	-
7	28.88	20.04	+
8	30.51	19.86	-
9	23.11	20.24	+
10	22.06	19.94	+
11	30.44	19.89	+
12	27.43	19.86	-
13	20.87	19.37	+
14	29.36	19.03	-
15	28.97	19.50	-
16	20.71	19.31	+
17	31.73	19.68	-
18	19.02	nt	+
19	28.12	20.09	-
20	23.85	20.06	+
21	28.97	20.39	-
22	26.47	19.52	-
23	22.17	20.29	+
24	26.22	20.05	-
25	29.84	20.25	-
26	20.85	20.03	-
27	29.23	19.27	-
28	27.55	20.23	+
29	21.06	20.50	+
30	29.48	20.10	-
31	29.33	19.39	-
32	24.05	21.64	-
33	25.94	19.35	-
34	24.72	20.72	+
35	23.60	19.91	+
36	31.56	19.86	-
37	21.07	20.38	+
38	20.86	19.22	-
39	26.30	19.59	-
40	21.57	20.55	-
